# Publisher Correction: A shear-dependent NO-cGMP-cGKI cascade in platelets acts as an auto-regulatory brake of thrombosis

**DOI:** 10.1038/s41467-018-07409-1

**Published:** 2018-11-20

**Authors:** Lai Wen, Susanne Feil, Markus Wolters, Martin Thunemann, Frank Regler, Kjestine Schmidt, Andreas Friebe, Marcus Olbrich, Harald Langer, Meinrad Gawaz, Cor de Wit, Robert Feil

**Affiliations:** 1 Interfakultäres Institut für Biochemie, University of Tübingen, 72076 Tübingen, Germany; 2 Institut für Physiologie, Universität zu Lübeck, 23562 Lübeck, Germany; 3 Physiologisches Institut, University of Würzburg, 97070 Würzburg, Germany; 4 University Hospital, Department of Cardiology and Cardiovascular Medicine, University of Tübingen, 72076 Tübingen, Germany; 5 Present Address: Division of Inflammation Biology, La Jolla Institute for Allergy and Immunology, 92037 La Jolla, CA USA

Correction to: *Nature Communications*; 10.1038/s41467-018-06638-8; published online 16 October 2018

The original version of this Article contained an error in the description of Supplementary Movie 4, in which the final sentence was inadvertently truncated. The HTML has been updated to include a corrected version of the ‘Description of Additional Supplementary Files’ file.

