# Supplementary material for: A shear-dependent NO-cGMP-cGKI cascade in platelets acts as an auto-regulatory brake of thrombosis
Source: Nat Commun. 2018 Oct 16;9:4301. doi: 10.1038/s41467-018-06638-8 (PMC6191445; doi:10.1038/s41467-018-06638-8)
Supplement: Supplementary file 1 — Supplementary Information [file 41467_2018_6638_MOESM1_ESM.pdf]

## SUPPLEMENTARY INFORMATION

# A shear-dependent NO-cGMP-cGKI cascade in platelets acts as an auto-regulatory brake of thrombosis

Lai Wen, Susanne Feil, Markus Wolters, Martin Thunemann, Frank Regler, Kjestine Schmidt, Andreas Friebe, Marcus Olbrich, Harald Langer, Meinrad Gawaz, Cor de Wit, Robert Feil

Supplementary Figures 1-11

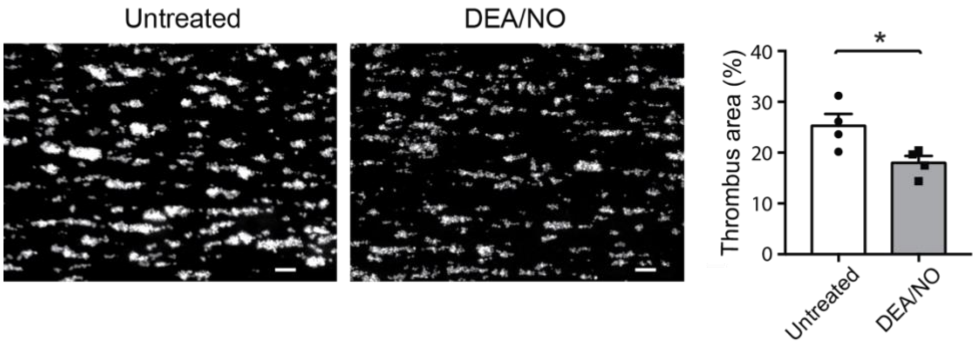


**Supplementary Figure 1. NO inhibits thrombus formation by human platelets under flow.** Human whole blood was labelled with $DiOC_6$ and perfused over a collagen-coated surface at a shear rate of 500 $s^{-1}$. Shown are representative images of thrombus formation in the absence (untreated) and presence of 10 μM DEA/NO. Scale bars, 50 μm. Thrombus formation was measured by the area covered by thrombi. Data are shown as mean ± SEM from 4 experiments; *P < 0.05 (Student`s *t*-test).

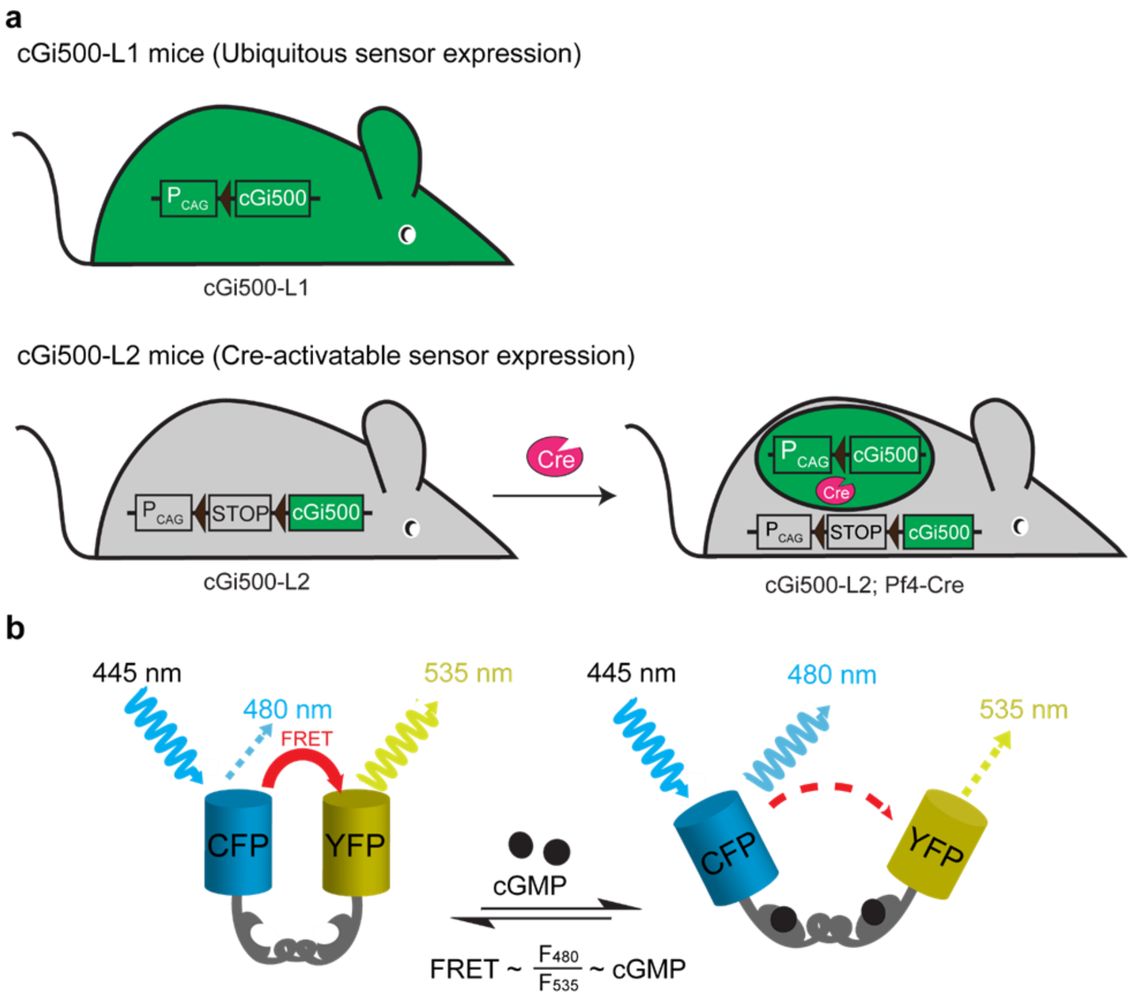


**Supplementary Figure 2. cGMP sensor mice for FRET-based cGMP imaging in platelets. a**, Schematic representation of cGMP sensor mice with global cGi500 expression (upper, *cGi500-L1*), and generation of platelet-specific cGi500-expressing mice by crossing the Cre/lox-activatable cGi500-L2 line to the platelet-specific Pf4-Cre line (lower, *cGi500-L2*; *Pf4-Cre*). **b**, Working principle of the FRET-based biosensor cGi500. cGi500 is a cGMP indicator consisting of the cGMP-binding domain of cGKI (grey) sandwiched between CFP and YFP. Upon excitation of CFP, part of the emitted energy is transferred via FRET to YFP, which is therefore also excited. When cGMP binds to the sensor, its FRET efficiency is decreased. Thus, the ratio of CFP over YFP emission (F480/F535) indicates the intracellular cGMP concentration.

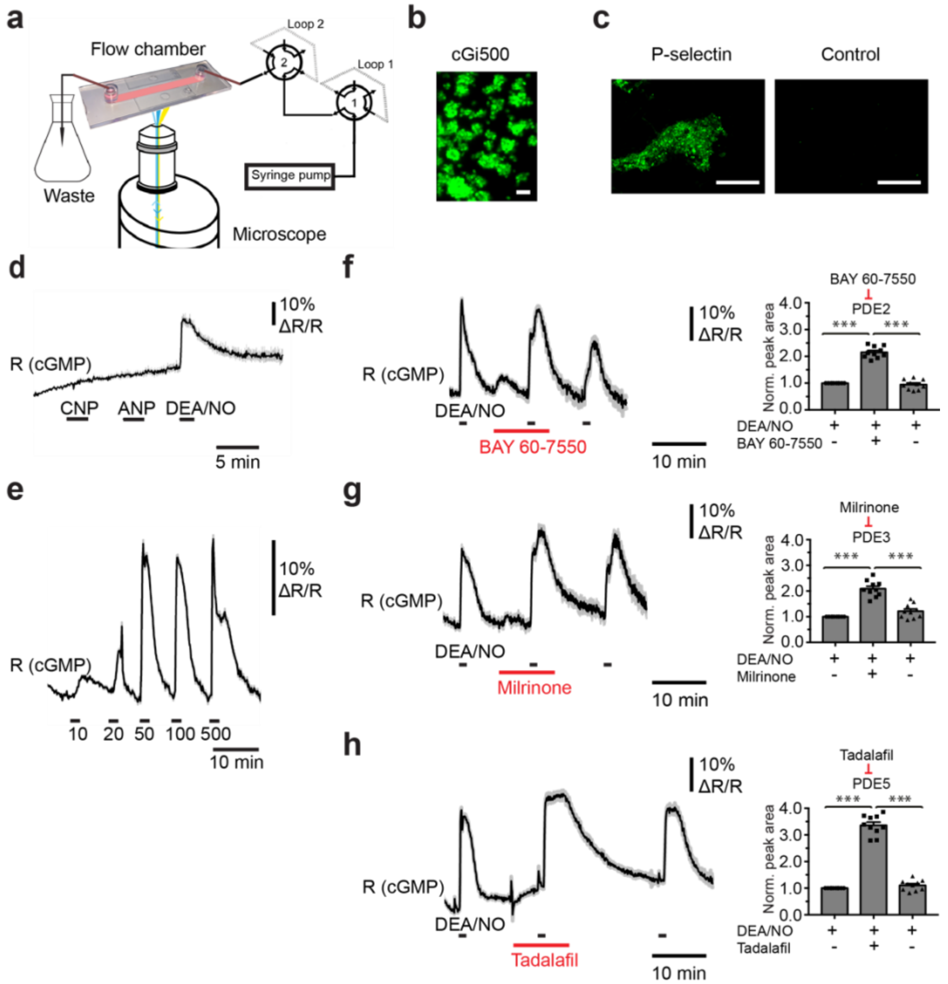


**Supplementary Figure 3. Characterization of cGMP signals in platelets. a**, Flow chamber setup for cGMP imaging in platelet thrombi *ex vivo*. **b**, Whole blood from a cGi500-L1 mouse was perfused at a shear rate of 500 $s^{-1}$ through a flow chamber resulting in thrombus formation on a collagen-coated coverslip. Sensor fluorescence was detected with an YFP filter set. Scale bar, 10 µm. **c,** Whole blood from a wild-type mouse was used to form thrombi on a collagen-coated coverslip and then stained with an antibody against P-selectin or only Alexa Fluor 488-conjuated secondary antibody (control). Scale bar, 10 µm. **d**, FRET/cGMP imaging was performed in platelet thrombi shown in (**b**). While superfusion with CNP (100 nM) or ANP (100 nM) did not increase platelet cGMP, exposure to DEA/NO (100 nM) led to a reversible cGMP increase in adhered platelets. **e**, DEA/NO concentration-response experiments with increasing concentrations of DEA/NO (in nM). **f-h**, PDE inhibition with (**f**) BAY 60-7550 (10 nM), (**g**) milrinone (10 µM) or (**h**) tadalafil (5 µM) potentiates DEA/NO (100 nM)-induced cGMP responses. Cells were stimulated three times with DEA/NO: first with DEA/NO alone, then a second time after pre-incubation of the thrombi with the respective PDE inhibitor, followed by wash-out of the PDE inhibitor and a third stimulation with DEA/NO alone. The specificity of each PDE inhibitor is indicated above each diagram (right). Peak areas were taken as a measure of the cGMP response and normalized to the first peak of each experiment. Data are shown as mean ± SEM (n = 10 thrombi); ***$P < 0.001$; *$P < 0.05$ (one-way ANOVA). Similar results were obtained in 2-3 independent experiments.

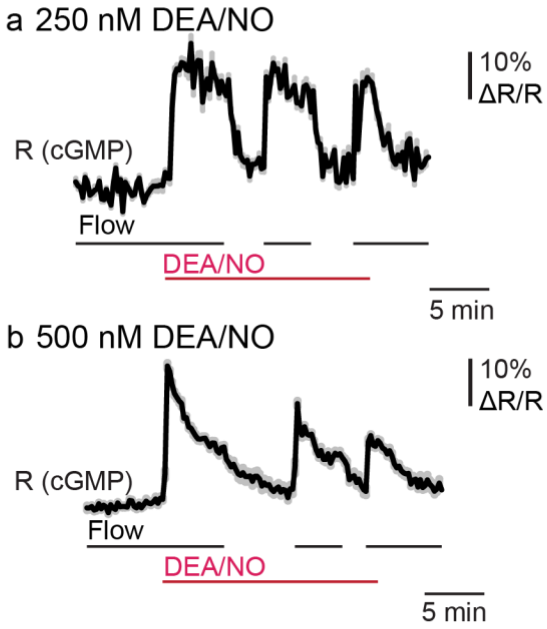


**Supplementary Figure 4. High concentrations of DEA/NO result in desensitization of mechanosensitive cGMP responses.** FRET/cGMP imaging was performed in cGi500-expressing platelet thrombi formed on collagen at a shear rate of 500 $s^{-1}$. Platelets were exposed to flow on/off conditions in the presence of (**a**) 250 nM and (**b**) 500 nM DEA/NO. Data are shown as mean ± SEM (n = 10 thrombi). Representative data from one of three independent experiments are shown.

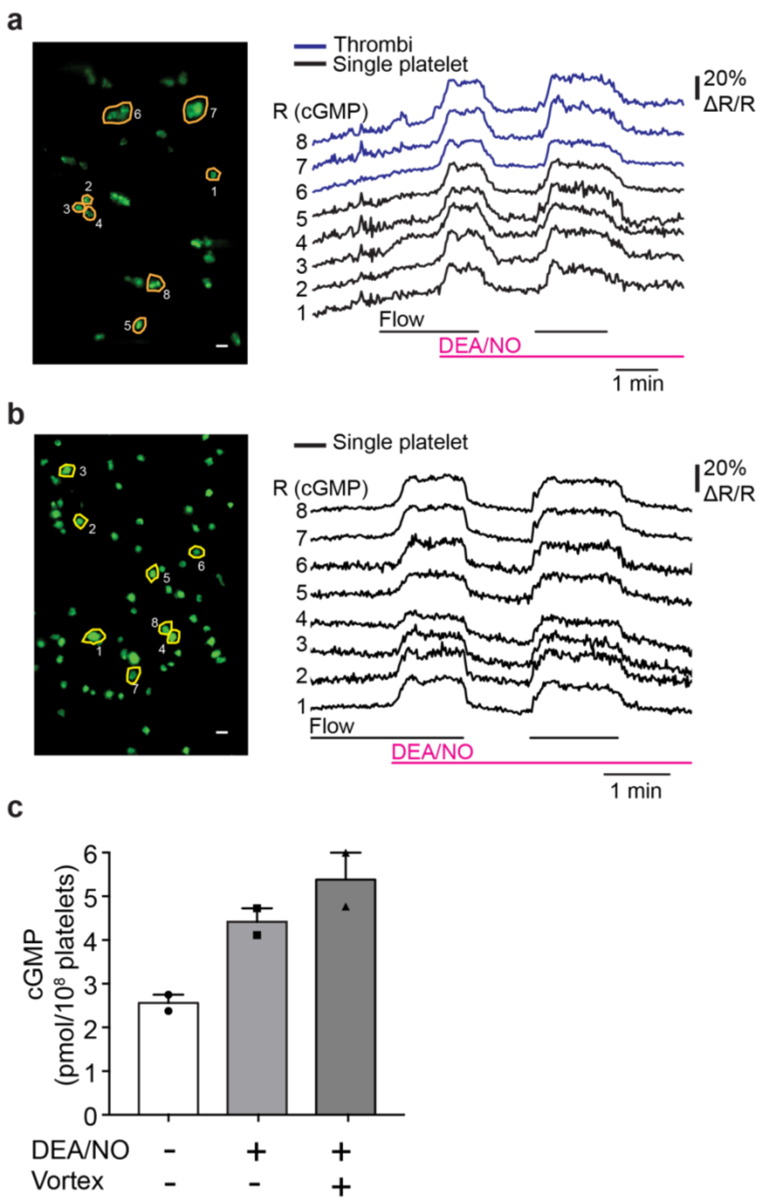


**Supplementary Figure 5. NO-induced cGMP responses in individual platelets are sensitive to shear stress**. **a**, **b**, Whole blood of cGi500-L1 mice was perfused at a shear rate of 500 $s^{-1}$ through flow chambers coated with (**a**) collagen or (**b**) fibrinogen. Platelets were exposed to flow on/off conditions in the presence of 100 nM DEA/NO. Representative data recorded from platelet thrombi (blue traces in **a**) or single platelets (black traces in **a** and **b**) are shown from one of three independent experiments. Numbers indicate the evaluated regions of interest as shown in the left images. Scale bars, 5 µm. **c**, Platelet suspensions isolated from wild type mice were incubated with 50 nM DEA/NO under static conditions (no vortexing) or on a vortex shaker for 15 s and then their cGMP content was determined by ELISA. Control platelets were allowed to rest in a tube without DEA/NO and vortexing. Data are obtained from duplicate measurements and shown as mean ± SEM.

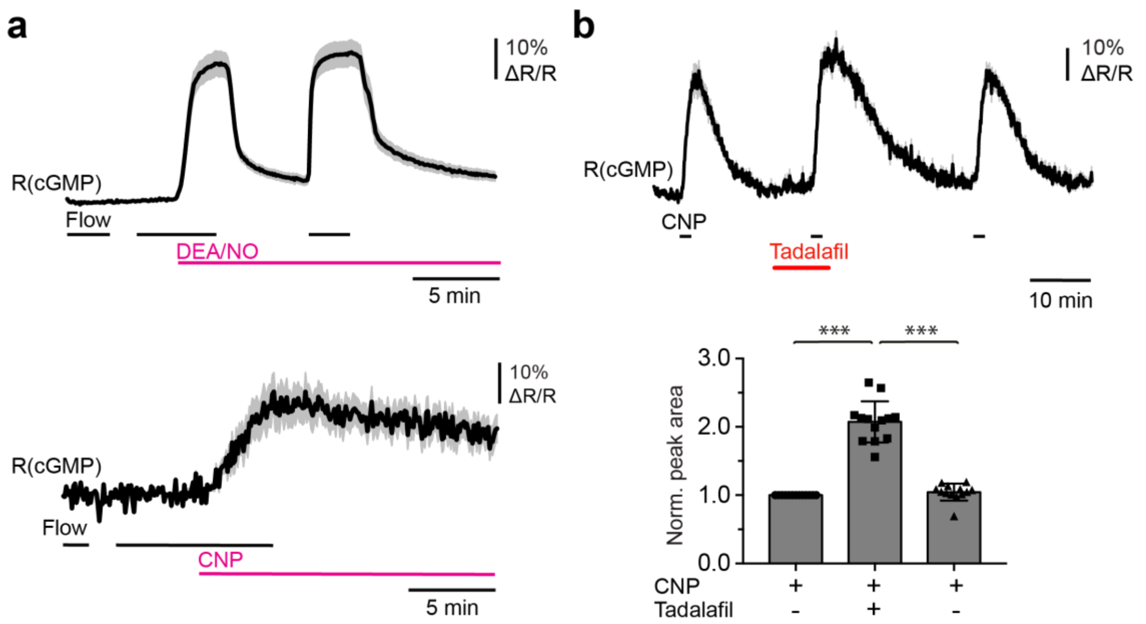


**Supplementary Figure 6. VSMCs also show a shear-sensitive NO-cGMP system. a**, Primary VSMCs isolated from mouse aortae were superfused at 500 $s^{-1}$ with 100 nM DEA/NO (upper) or 50 nM CNP (lower) and subjected to on/off flow conditions. Data are shown as mean ± SEM (n=8 VSMCs). **b**, PDE5 inhibition with tadalafil (100 nM) potentiated the CNP-induced cGMP response in VSMCs. Cells were continuously superfused at 500 $s^{-1}$ and stimulated three times with CNP: first with CNP alone, then a second time after pre-incubation of the cells with tadalafil, followed by wash-out of the PDE inhibitor and a third stimulation with CNP alone. Peak areas were taken as a measure of the cGMP response and normalized to the first peak of each experiment. Data are shown as mean ± SEM (n=13 VSMCs); ***P < 0.001 (one-way ANOVA).

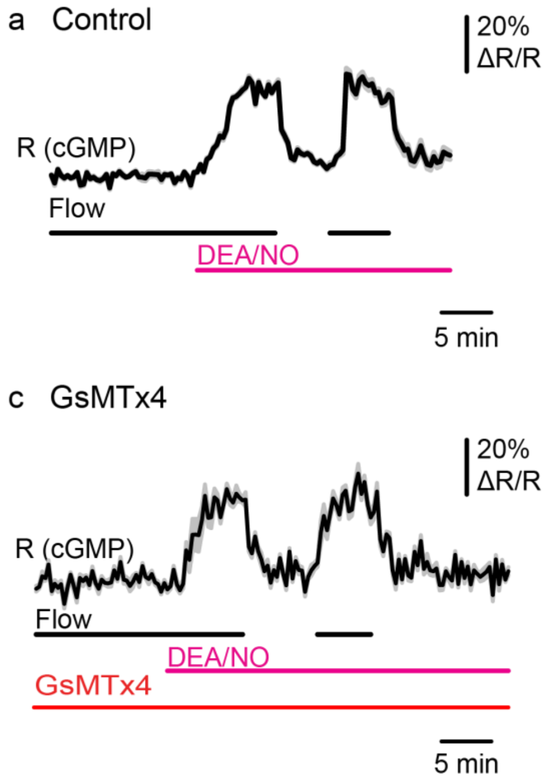


**Supplementary Figure 7. Inhibition of mechanosensitive ion channels or β$_3$ integrin does not affect shear-regulated cGMP signals.** FRET/cGMP imaging was performed in cGi500-expressing platelet thrombi formed on collagen at a shear rate of 500 $s^{-1}$. Platelets were exposed to flow on/off conditions in the presence of of 100 nM DEA/NO under (**a**) control conditions, or after pre-incubation with (**b**) 1 µM Pyr3 (inhibitor of TRPC3), (**c**) 500 nM GsMTx4 (nonselective inhibitor of TRPC1, TRPC6, Piezo1, and stretch-activated cation channels), or (**d**) 10 µg/mL anti-β$_3$ integrin antibody. Data are shown as mean ± SEM (n = 14 thrombi). Representative data from one of three independent experiments are shown.

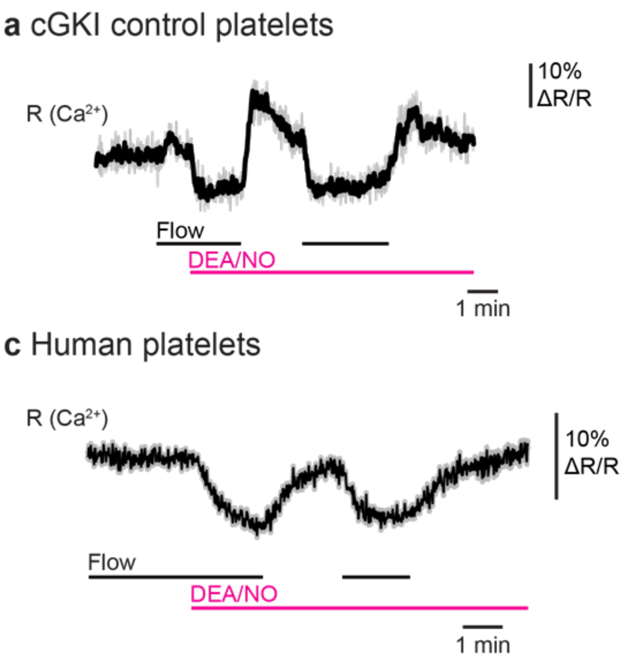


**Supplementary Figure 8. Flow-regulated NO/cGMP-$Ca^{2+}$ crosstalk is mediated by cGKI and is also present in human platelets**. **a**, **b**, $Ca^{2+}$ imaging was performed in platelet thrombi formed *ex vivo* on a collagen-coated surface. Thrombi from (**a**) control mice (*SM22*$^{+/I\beta}$; *cGKI*$^{+/L-}$) or (**b**) SM-Iβ rescue mice lacking cGKI in platelets (*SM22*$^{+/I\beta}$; *cGKI*$^{L-/L-}$) were superfused at 500 $s^{-1}$ and exposed to on/off flow conditions in the presence of 100 nM DEA/NO. **c**, $Ca^{2+}$ imaging was performed in human platelet thrombi formed on a collagen-coated surface. Flow (500 $s^{-1}$) was switched off and on in the presence of 100 nM DEA/NO. Data are shown as mean ± SEM (n = 10 thrombi). Results are representative of three independent experiments.

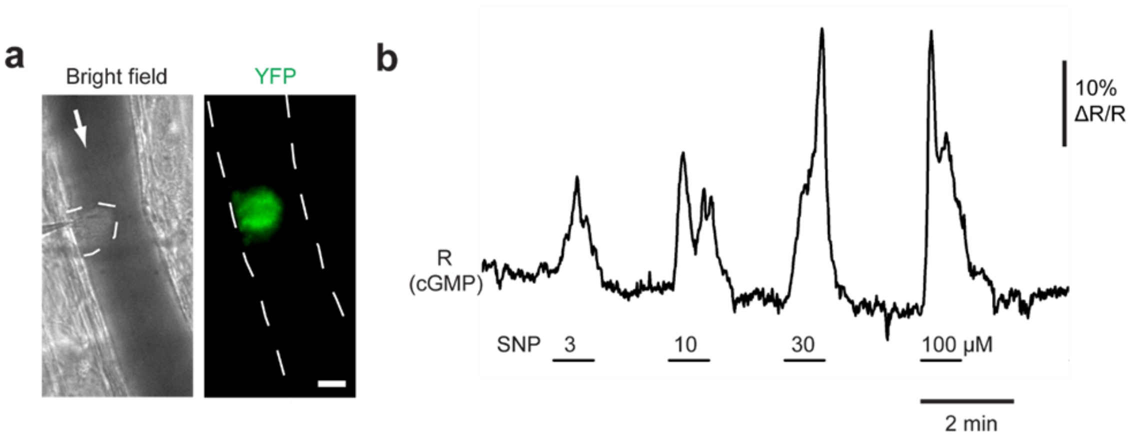


**Supplementary Figure 9. FRET-based intravital cGMP imaging in a thrombosis model using epifluorescence microscopy. a**, The cremaster microcirculation of platelet-specific cGMP sensor mice (*cGi500-L2*$^{fl/fl}$; *Pf4-Cre*$^{tg/+}$) was visualized using an upright epifluorescence microscope. Thrombus formation was induced by mechanical injury of an arteriole with a micropipette and monitored under bright field and fluorescence (YFP filter set) illumination. **Left**, White arrow and broken line indicate the direction of blood flow and the thrombus, respectively. **Right**, Broken line delineates the vessel wall. Platelets are green. Scale bar, 20 µm. **b**, Representative recording of FRET/cGMP changes in platelet thrombi in response to increasing concentrations (indicated in µM) of the NO-releasing agent sodium nitroprusside (SNP).

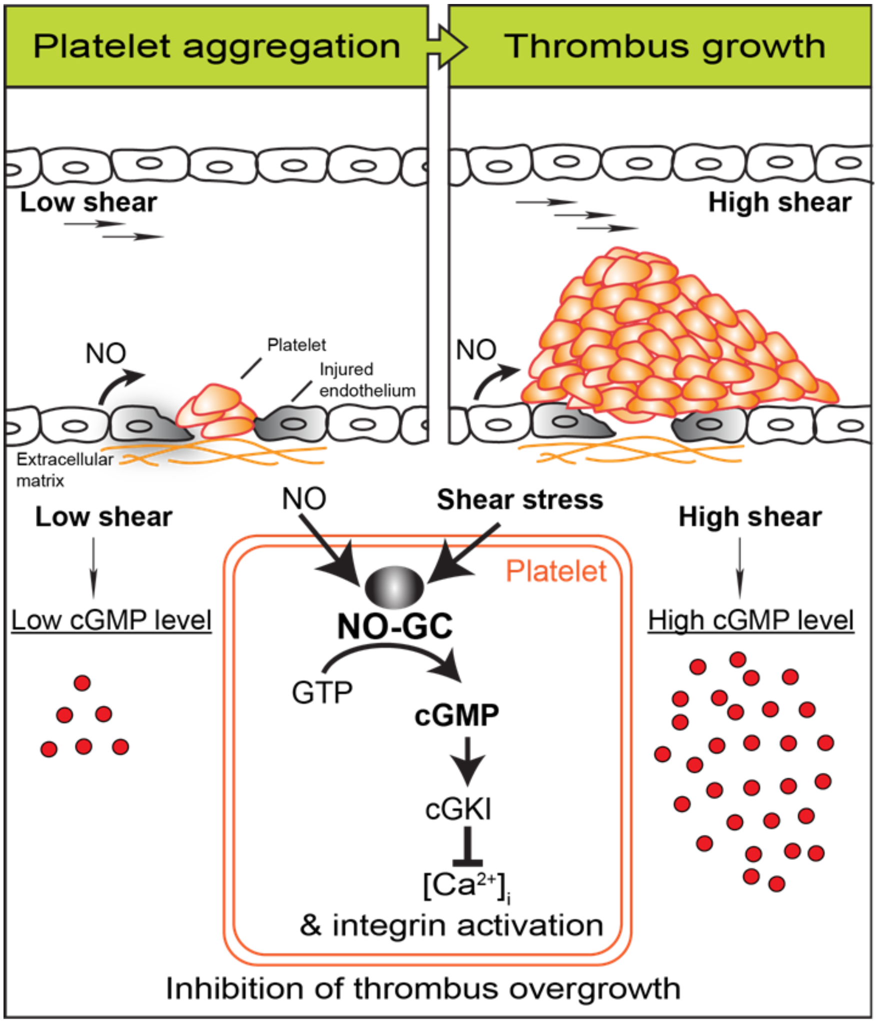


**Supplementary Figure 10. Model of a shear-dependent cGMP brake that prevents thrombus overgrowth, but leaves primary hemostasis intact.** Shear stress sensitizes platelets to cGMP generation in response to NO released from the vascular endothelium. Initially, aggregated platelets are exposed to low shear stress, keeping the platelet cGMP concentration low and allowing efficient initiation of thrombus formation (left). During growth of the thrombus, platelets at the periphery are increasingly exposed to shear stress leading to an increased cGMP concentration in these platelets (right), activation of cGKI and subsequent reduction of the intraplatelet $Ca^{2+}$ concentration ($[Ca^{2+}]_i$) and inhibition of integrin activation. This shear-dependent NO-cGMP-cGKI cascade facilitates thrombus dissolution and limits thrombus growth. Note that the cGMP thrombosis brake does not inhibit the formation of primary hemostatic plugs, which form in the vessel wall and extravascular space under low shear conditions (not shown), thus, minimizing bleeding risk during cGMP-based anti-thrombotic therapies.

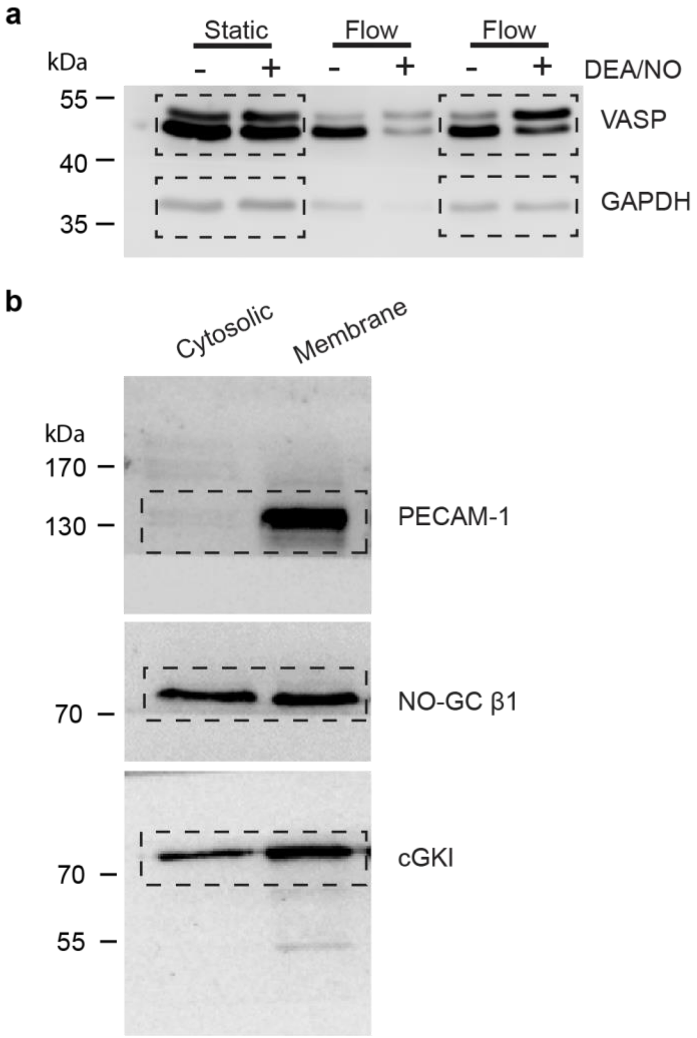


**Supplementary Figure 11. Uncropped western blots for the gels shown in Figure 1c (a) and Figure 2h (b).** Note that blot membranes were cut into strips in order to probe for multiple proteins. The dotted rectangles indicate regions shown in Figure 1c and Figure 2h.
