# Supplementary material for: A shear-dependent NO-cGMP-cGKI cascade in platelets acts as an auto-regulatory brake of thrombosis
Source: Nat Commun. 2018 Oct 16;9:4301. doi: 10.1038/s41467-018-06638-8 (PMC6191445; doi:10.1038/s41467-018-06638-8)
Supplement: Supplementary file 7 — Description of Additional Supplementary Files [file 41467_2018_6638_MOESM7_ESM.docx]

**Title:** Supplementary Movie 1.
**Description:** Shear-dependent cGMP signals in platelet thrombi exposed to NO in a flow chamber ex vivo. Thrombi formed from cGi500-expressing platelets in a collagen-coated flow chamber were superfused at a shear rate of 500 s^-1^. FRET/cGMP signals were recorded by epifluorescence microscopy. In the FRET ratio movie, brighter colors indicate higher cGMP concentrations. Addition of DEA/NO (100 nM) to the buffer led to an increase of the cGMP signal. Then flow was repeatedly switched off and on in the presence of DEA/NO resulting in a cGMP decrease and increase, respectively. Scale bar is 20 µm.

**Title:** Supplementary Movie 2.
**Description:** Endogenous cGMP signals in mechanical injury-induced platelet thrombi in vivo. Thrombosis was induced by mechanical injury of a cremaster arteriole of a platelet-specific cGi500 mouse, and then observed by intravital imaging with a confocal spinning disk microscope. Thrombus growth was monitored by recording YFP emission of the sensor (left). The cGMP level in platelets was simultaneously monitored by FRET ratio imaging, with brighter colors indicating higher cGMP concentrations (right). The broken line delineates the vessel wall. The direction of blood flow was from top to bottom. Note that cGMP concentrations were higher in the shear-exposed periphery of the thrombus than in its core region. Scale bar is 20 µm.

**Title:** Supplementary Movie 3.
**Description:** Laser-induced arterial thrombosis in the presence and absence of platelet NO-GC. Thrombosis was triggered by laser-induced injury of a cremaster arteriole of a control mouse (left) or a platelet-specific NO-GC β1 knockout mouse (right) expressing the cGi500 sensor in platelets. Thrombus growth was monitored by recording YFP emission of the sensor by intravital imaging with a confocal spinning disk microscope. The broken line shown at the beginning of the movie delineates the vessel wall. The direction of blood flow was from top to bottom and is indicated by an arrow. Note that stabilization of the NO-GC-deficient thrombus takes longer and the size of the stabilized thrombus at the end of the experiment is bigger than that of the control thrombus. Scale bar is 20 µm.

**Title:** Supplementary Movie 4.
**Description:** Endogenous cGMP signals in laser-induced platelet thrombi in vivo. Thrombosis was triggered by laser-induced injury of a cremaster arteriole of a control mouse (left) or a platelet-specific NO-GC β1 knockout mouse (right) expressing the cGi500 sensor in platelets. The cGMP level in platelets was simultaneously monitored by FRET ratio imaging, with brighter colors indicating higher cGMP concentrations. Note that the movies in the control mouse (left) and platelet-specific NO-GC β1 knockout mouse (right) were evaluated in parallel and with the same color scale bar as in Figure 3h. The broken line delineates the vessel wall. The direction of blood flow was from top to bottom. Scale bar is 20 μm.
